# Academy of Stomatology

**Published:** 1897-08

**Authors:** J. Howard Gaskill

**Affiliations:** Secretary


### ACADEMY OF STOMATOLOGY.

A regular meeting of the Academy of Stomatology was held March 23, 1897, at the rooms of the society, 1731 Chestnut Street, Philadelphia, the President, Dr. James Truman, in the chair.

Dr. H. C. Register, chairman of the Clinic Committee, made a report, including his "Method of Operating."

Dr. W. A. Capon gave a report of his clinic, exhibiting the preparation of a "porcelain jacket," as follows:

The case presented was a lady with deformed lateral incisors, commonly called "pig" or "rice" teeth, perfectly sound, with pulp healthy; the bite unnatural through extreme prognathism; the lower incisors closing completely outside of the upper, thereby enabling me to give undivided attention to the crown alone. With such cases very little preparation of the tooth is required. Simply grinding both lingual and labial surfaces slightly flatter and short enough to leave room for thickened cutting-edge of porcelain. After this procedure the circumference of the tooth was taken with wire, the same as for gold cap, and the size cut from platinum plate, gauge 30, and bent in tubular form, joints lapped and soldered with pure gold. This tube was fitted to extend slightly under free margin of gum, and should be quite as long as the tooth. While the tube was in position I marked the outline of adjoining teeth on its surface to indicate the final shape. The lingual portion was ground to this line and then reinforced by soldering another piece of platinum of the same gauge to that surface. The labial surface was then ground sufficiently thin to allow of burnishing to the tooth, reducing the tube to a hollow wedge. This part of the operation is quite the most important and, I may say, the most difficult to perform, for on it depends the future of the crown and probably the operator's reputation with that particular patient. The platinum frame was then placed in position and a thin porcelain veneer adjusted on its surface and held by porcelain body. The whole was then gently drawn from the tooth and dried, ready for the furnace.

After fusing, the crown was tried in its place and found slightly prominent, which fault was easily remedied by grinding the surface. More body was then added to overcome the shrinkage of first baking and to give the crown its proper shape, then fired for the second time, giving the porcelain a uniform density and surface, restoring the original gloss destroyed by grinding.

After polishing the metal portion the crown was cemented in position, thus completing a crown natural in appearance, durable, and extremely applicable in this instance. No other crown can *artistically* overcome such deformities and still retain original form of the tooth, with pulp intact, unless it would be a gold cap,—and would that kind of an operation be a remedy?

The platinum was ground to form instead of cutting, so that a better joint is secured. The great heat in fusing would melt the solder from a flush joint and leave a crevice when finished. The same reason is advanced for lapping the joint when making tubes.

The furnace used was the Land "midget," one of the original gas furnaces, and still unexcelled for quickness and convenience, requiring only five minutes to fuse continuous gum body from time of lighting.

Dr. H. E. Roberts gave a clinic on the bleaching of teeth by the use of pyrozone, aided by cataphoresis. The case was a lady with both centrals and the left lateral incisor badly discolored, the teeth having large approximal cavities. After having removed all decay and wiped out the cavities with a fifty-per-cent. solution of sulphuric acid, neutralizing the acid with bicarbonate of soda, three pieces of platinum wire were twisted together, allowing one end of each to be loose; these were put in the canals and tied there so that the bleaching could be carried on in all three at once. Then a twenty-five-per-cent. ethereal solution of pyrozone was diluted with water, the ether was allowed to evaporate, and the aqueous solution which remained was used to saturate the cotton which had been placed in the canals, the current turned on and gradually brought up to thirty volts, being kept there for one hour, when the lateral was found to be so much improved that the wire from it was taken out and placed in the other side of the left central, making two contacts in that tooth. This was allowed to remain about one-half hour longer, when the electric current was cut off. Cotton saturated with the pyrozone solution was then sealed in the teeth and allowed to remain for two days, when the teeth still showed a greater improvement.

Dr. Gilliams paper on "Some Dental Manifestations of Gout" followed.

(For Dr. Gilliams's paper, see page 441.)

DISCUSSION.

Dr. Darby stated that he had seen so many similar cases that it seemed to him pyorrhœa must be associated with the gouty diathesis, although in some cases of calcic pericementitis and erosion gout is not evident, but there seems to be a strong probability of manifesting itself at a later period; also in very aggravated forms of gout there may be an entire absence of dental trouble, as an instance, a lady who was suffering from an inherited case of gout, and who for years had been compelled to be under a rigid anti-gout treatment with but partial relief; but, fortunately, she was entirely free from pyorrhœa alveolaris.

*Dr. Register.*—I believe pyorrhœa to be a manifestation of the gouty diathesis, having had personal experience in the matter, entailing the loss of a lateral incisor; although most authorities credit the young with being free from pyorrhœa. I have a patient, fifteen years of age, presenting that condition, and whose family history shows gouty tendencies, his father having died from rheumatic fever.

*Dr. Roberts.*—Gout may manifest itself in many ways, and although there may be no other symptoms than that of pyorrhœa, I believe some time there will be other indications of a gouty condition.

*Dr. Jack.*—I would like to ask if any one can assign a cause for the receding of the gums and the absorption of the alveolar process when there is no evidence of a calcic deposit or inflammation of the parts?

*Dr. Truman.*—There is apparently a condition frequently met with, and as often overlooked, that seems to answer the description of Dr. Jack's query. It is recession of the gums without any of the symptoms of pyorrhœa.

This is not confined to old age, but may present at a comparatively early period, from thirty to thirty-five. In a case coming under his care, at the latter age, all the teeth were more or less affected by resorption without any of the ordinary signs of pyorrhœa. On examination, microscopically, of one of the teeth thrown out by the destructive degeneracy of the tissues, it exhibited a nearly complete eradication of the tubulated structure. So marked was this that the section presented the appearance of a transparent plate of glass. The question of calcification of the inner tubular tissue was then in warm dispute, and as it was difficult to determine the cause of the obliteration of the tubuli, it was left then without explanation. The only reasonable conclusion, however, is that the teeth had become practically foreign bodies, and were thrown out as organs incapable of proper nutrition. Senile dentine—dentine of old age—may and doubtless does produce the same effect.

*Dr. William H. Trueman.*—There is always a tendency for teeth to assume their proper position in the arch; so I want to particularly commend Dr. Roberts for his remarks about waiting until we are sure nothing further can be gained by delay. The tendency of teeth to return to their former position is a very serious condition we must recognize in regulating, and unless they can be fixed in the new position our labor will be lost.

*Dr. Register.*—I want to commend Dr. Roberts for the valuable suggestion he has given us in his method of regulating; it is unique, simple, easily constructed, and efficient.

*Dr. Gardiner.*—I have seen most of the cases of Dr. Roberts for the past seven years, and I cannot but express my pleasure in the way he handles his cases of regulating.

Subject passed.

Adjourned.

J. Howard Gaskill,
*Secretary.*

